# Dual scale light weight cross attention transformer for skin lesion classification

**DOI:** 10.1371/journal.pone.0312598

**Published:** 2024-12-06

**Authors:** Dhirendra Prasad Yadav, Bhisham Sharma, Shivank Chauhan, Julian L. Webber, Abolfazl Mehbodniya

**Affiliations:** 1 Department of Computer Engineering & Applications, G.L.A. University, Mathura, Uttar Pradesh, India; 2 Centre for Research Impact & Outcome, Chitkara University Institute of Engineering and Technology, Chitkara University, Rajpura, Punjab, India; 3 Department of Electronics and Communication Engineering, Kuwait College of Science and Technology (KCST), Doha Area, Doha, Kuwait; UPES Dehradun, INDIA

## Abstract

Skin cancer is rapidly growing globally. In the past decade, an automated diagnosis system has been developed using image processing and machine learning. The machine learning methods require hand-crafted features, which may affect performance. Recently, a convolution neural network (CNN) was applied to dermoscopic images to diagnose skin cancer. The CNN improved its performance through its high-dimension feature extraction capability. However, these methods lack global co-relation of the spatial features. In this study, we design a dual-scale lightweight cross-attention vision transformer network (DSCATNet) that provides global attention to high-dimensional spatial features. In the DSCATNet, we extracted features from different patch sizes and performed cross-attention. The attention from different scales improved the spatial features by focusing on the different parts of the skin lesion. Furthermore, we applied a fusion strategy for the different scale spatial features. After that, enhanced features are fed to the lightweight transformer encoder for global attention. We validated the model superiority on the HAM 10000 and PAD datasets. Furthermore, the model’s performance is compared with CNN and ViT-based methods. Our DSCATNet achieved an average kappa and accuracy of 95.84% and 97.80% on the HAM 10000 dataset, respectively. Moreover,the model obtained 94.56% and 95.81% kappa and precision values on the PAD dataset.

## 1. Introduction

A skin lesion refers to an abnormal growth on the skin that is different from the typical properties of the surrounding skin [1]. There are two categories of skin lesions: primary and secondary. Primary wounds are atypical skin disorders that may arise gradually or be congenital. In addition, skin lesions can arise due to the worsening or modification of primary skin lesions [2, 3]. When a mole is abraded to the point of bleeding, a crust forms, resulting in a second type of skin lesion. Expert dermatologists recommend three solutions for affected skin based on the nature of the lesion: self-care, medicine, or surgical intervention. Despite their benign appearance [4], several types of skin lesions can pose a significant concern to patients as they might indicate the existence of cancer and necessitate surgical removal. In all types of skin lesions, melanoma is the most severe form of skin cancer and becomes deadly once it metastasizes. Nevertheless, it can be effectively treated if detected in its first stage [5]. Therefore, it is crucial to accurately diagnose skin lesions to protect patients’ growth and ensure that they receive immediate medical attention.

Visual examination of human skin with the naked eye is complex and requires a highly skilled dermatologist. Dermoscopy is a noninvasive imaging technique for diagnosing melanoma [6]. However, in conventional clinical cases, dermatologists can correctly detect melanoma, but accuracy could be better. Therefore, studies have focused on developing methods to help clinicians distinguish between melanoma and benign tumours to save patients’ lives. Machine learning methods utilized the morphological characteristics of the skin lesion categorization [7]. In the HAM 10000 dataset, skin lesions are classified into seven categories. In contrast, the PAD dataset has six types of skin lesions. The experts manually extracts the texture, shape and colour features of skin lesions and feeds them to the machine learning algorithms for classification [8]. This method has improved the classification accuracy compared to the conventional approach. However, method performance is dependent on the expertise of the expert.

Recently, CNN methods have been widely used in skin lesion diagnosis [9, 10]. The CNN method overcomes the challenges imposed by the handcrafted features-based method through automatic high-dimension feature extraction capabilities. However, designing a low-cost CNN model is a difficult task. In addition, classical CNN methods lack global attention to the spatial features of the skin lesion [11]. This makes performance less reliable.

In short manual inspection of the skin cancer is time consuming and expert dependent, Whereas, machine learning based method require hand-crafted features for the training of the algorithm, which is prone to the error. Moreover, CNN based method extracts shallow spatial features for the training of the model, due to this performance is not optimal in several applications.

In this study, we proposed a DSCATNet (dual-scale lightweight cross-attention vision transformer network) in which input image is divided into 8x8 and 16x16 pixels patches for and fed to the dual-scale spatial feature extraction module for the training of the model. Furthermore, a cross-attention module is utilized to focus on the relevant region of the skin lesion. In addition, a lightweight transformer encoder is utilized to provide global context to the high-dimensional spatial features and Softmax layer for the classification of the skin cancer. Quantitative results of the DSCATNet on the HAM 10000 and PAD datasets were compared with those of CNN and ViT-based methods.

The major contribution of the manuscript is as follows.

(a) We design a dual-scale feature extraction module to capture coarse and fine details of the objects with different shapes and sizes in the skin lesions.

(b) Cross-attention across two scales effectively integrates information and allows queries from one scale to interact with keys and values from the second scale. This gives the model a clearer understanding of spatial information.

(c) We applied a fusion strategy for the dual-steam cross attention and integrated it with the transformer to capture long-range dependency and global context to the features.

(d) The model performance is evaluated on HAM 10000 and PAD datasets and compared with the CNN and ViT-based methods.

The remainder of the manuscript is as follows.

In section 2, the detailed literature survey of several skin lesion methods is presented, Whereas the proposed method architecture and algorithm are presented in section 3. The dataset description and experimental results are elaborated in section 4. Furthermore, a detailed discussion and ablation study is available in section 5. Finally, we conclude the proposed methods in section 6.

## 2. Related work

Talavera et al. [12] proposed a skin lesion dataset containing 615 images. Furthermore, they designed a lightweight ten-layer CNN model inspired by VGG16 and compared performance for symmetric classification of skin lesions with a transfer learning-based approach. Ding et al. [13] suggested Deep Attention Branch Networks (DABN), which add attention branches to Deep Convolutional Neural Networks (DCNN) for the diagnosis of lesions more accurately. Their model was versatile and introduced entropy-guided loss weighting (ELW) to address dataset class imbalances. Qian et al. [14] proposed a grouping of multi-scale attention blocks (GMAB) for multi-scale feature extraction to improve lesion diagnosis. Further, they utilized class-specific loss weighting to handle category imbalances. Iqbal et al. [15] developed CSLNet (Skin Lesions Network), which had 54 convolution layers. Their model contained four kernel units. Furthermore, model performance was evaluated on the three datasets. The CSLNet achieved precision and sensitivity of 94% and 93% in ISIC-17.

Rahman et al. [16] compared the performance of the five ensemble CNN model for classifying seven skin lesion types. They evaluated model performance on 18,730 dermoscopy images fromISIC 2019 and HAM10000 datasets. Furthermore, model performance was tested after employing techniques like class balancing, noise removal, and data augmentation. Calderón et al. [17] developed bilinear architecture using ResNet 50 and VGG16 to classifyskin lesions in the HAM 10000 dataset. In addition,a data augmentation technique was used to address data imbalances. Pratiwi et al. [18] designed an ensemble model using ResNet50 and Inception V3 to classify seven types of skin lesions in the HAM 10000 dataset and achieved a classification accuracy of 89.90%. In another research, Saeed and Zeebaree et al. [19] developed an automated system for the diagnosis of skin lesions. Their method utilized DCNN (Deep convolution neural network) and transfer learning techniques to enhance the classification performance. In addition, data augmentation techniques were used to imitate the training sample. Hosny et al. [20] developed an AI (artificial intelligence) based explainable model for skin lesion classification. They captured the features using inherent block, and max-pooling was used to reduce the dimension of the feature map. The explainable AI method obtained 92.89% classification accuracy on the HAM 2018 dataset.

Mahbod et al. [21] performed a transfer learning-based comparative study of the different CNN methods, including SeReNeXt-50, EfficientNetB1 and EfficientNetB0, on the ISIC2018 dataset. In addition, a muscle multi-CNN was designed and achieved an accuracy of 86.2%. Thurnhofer-Hemsi et al. [22] proposed an enhanced convolutional neural network and a test-time shifting model for classifying skin lesions. The shifting model generated multiple replicas of the test image by shifting it regularly, then feeds them into the ensemble-based classifiers. The combined outputs from these classifiers determined the final classification. Afza et al. [23] developed a hierarchical framework for skin lesion analysis, integrating superpixels and deep learning. Enhanced contrast in dermoscopy images precedes lesion segmentation using superpixels. The segmented lesions were mapped onto images for feature extraction by utilizing ResNet-50. The classification of the skin lesion was performed using Naïve Bayes.

Hoang et al. [24] segmented the skin lesion region followed by wide-ShuffleNet was trained and validated on the ISIC 2019 and HAM 10000 datasets. Khan et al. [25] performed skin lesion classification on the images obtained through two modules. Their method combined two types of images for localization: HDCT-based saliency segmentation and binary images from a convolutional neural network. After that, performance was improved by maximal mutual information to produce segmented RGB (Red, Blue Green) lesion images. Furthermore, a pre-trained DenseNet201 model extracts features in the classification module. These features’ dimensions were reduced via t-SNE and fused using MCCA (multi canonical correlation) before classification by a multi-class ELM (extreme learning machine) classifier. Villa-Pulgarin et al. [26] performed skin lesion classification on the HAM10000 dataset. They compared the performance of the three CNN models, DenseNet-201, Inception-ResNet-V2, and Inception-V3. Sevli [27] proposed a CNN-based model for classifying seven skin lesions. After integrating it into a web app, the dermatologists evaluated it in two phases. Phase one affirmed the model’s diagnostic ability, whereas phase two demonstrated its capability to rectify expert misdiagnoses, highlighting the utility of computer-aided systems in skin lesion diagnosis. Shetty et al. [28] proposed a CNN-based model for detecting skin malignancy. Using a subset of the HAM10000 dataset with augmentation, their method achieved 95.18% accuracy.

Afza et al. [29] classified skin lesions using feature fusion and extreme machine learning techniques. This method utilized techniques such as image acquisition, deep feature extraction, feature selection, feature fusion, and classification. Popescu et al. [30] proposed a skin lesion classification system using CNN and collective intelligence. They trained the CNNs on the HAM10000 dataset and predicted seven types of skin lesions. Kassem et al. [31] classified skin lesions using a pre-trained GoogleNet. Parameters were initialized with pre-trained values and adjusted during training. The GoogleNet classified eight skin lesion classes: melanoma, melanocytic nevus, basal cell carcinoma, actinic keratosis, benign keratosis, dermatofibroma, vascular lesion, and squamous cell carcinoma in the ISIC2019 dataset. Their model achieved a classification accuracy and precision of 94.92% and 80.36%, respectively. Some recent skin lesion diagnosis methodsare summarized in Table 1.

**Table 1:** Summary of the recent methods for skin lesion.

Study	Dataset	Model	Accuracy (%)
Hosny et al. [20]	HAM10000	Deep inherent learning method	92.89
Shivakumar et al. [32]	ISIC 2019	ResNet50	94.00
Kaur and Kaur et al. [33]	ISIC 2019	Weighted Sum method (WSM)	99.01
Fayyad et al. [34]	HAM10000	Uncertainty quantification methods	90.00
Su et al. [35]	HAM10000	Generative Adversarial Networks (GANs)	98.23
Khan et al. [36]	HAM10000	CNN	87.02
Wei and Ji et al. [37]	7-point Checklist database	Multi-modal bilinear fusion with hybrid attention mechanism (MBF-HA)	76.3
Adebiyi et al. [38]	HAM10000	Multimodal deep learning	94.11

We design a dual-scale feature extraction module to capture coarse and fine details of the objects with different shapes and sizes in the skin lesions. Existing multiscale attention-based models treat each scale independently; due to this, they miss the inter-scale interaction and crucial contextual information. In addition, we designed a cross-attention module across two scales that effectively integrates information and allows queries from one scale to interact with keys and values from the second scale. This gives the model a clearer understanding of spatial information. At the same time, classical ViT operates on a single scale and performs self-attention, which makes it less practical for diverse spatial features. Furthermore, we applied a fusion strategy for the dual-steam cross attention and integrated it with the transformer to capture long-range dependency and global context to the features. Kaur et al. [33] utilized a fusion of DenseNet-201, ResNet-152 and Squeeznet to classify ISIC2019 dataset images into melanoma and non-melanoma. In addition, they first segment the affected region of the skin lesion. After that, data augmentation techniques are applied to increase the size of the dataset to 17888 images. Furthermore, they trained and tested on the augmented dataset to evaluate the performance. Meanwhile, Su et al. [35] classify the HAM 10000 dataset images using ResNet-50. To increase the size of the dataset, they utilized StyleGAN and generated 18500 synthetic images. After training, they compared their model performance with the other methods. The proposed study evaluated the DSCATNet performance on the HAM 10000 and PAD datasets. In the HAM 10000 dataset, 10015 images are present for the training and validation. We do not perform data augmentation techniques to increase the dataset size of the HAM 10000. Moreover, a 5-fold cross-validation scheme is applied to evaluate the model performance. The performance measures are an average of 5fold in the proposed approach. In addition, the proposed method’s computation cost is comparatively less compared to Kaur et al. [33] and Su et al. [35].

## 3. Methodology

In this section, we present the datasets and proposed method description.

### 3.1. Dataset

The HAM 10000 dataset contains 10015 dermatoscopic images with a resolution of 600x450 pixels and is stored in.Jpeg format. These images are divided into seven categories: actinic keratoses (AKIEC), basal cell carcinoma (BCC), benign keratosis-like lesions (BKL), dermatofibroma (DF), melanoma (MEL), melanocytic nevi (NV) and vascular lesions (VASC). The AKIEC, BCC, BKL, DF, MEL, NV, and VASC have 327, 514, 1099, 115, 1113, 6705 and 142 images, respectively [39].

The PAD dataset contains 1612 images stored in Jpeg format with varying resolution. In the dataset, images are categorized into six classes, including Carcinoma (BCC), Actinic Keratosis (ACK), Nevus (NEV), Basal Cell, Melanoma (MEL), Seborrheic Keratosis (SEK), and Squamous Cell Carcinoma (SCC). The ACK, BCC, MEL, NEV, SCC, and SEK have 543, 442, 67, 196, 149,and 215 images, respectively [40]. This dataset has fewer images in each class, which may cause the mode to overfit. Therefore, we applied the data augmentation technique, horizontal and vertical flips, and increased the dataset size five times. After data augmentation, we have 8060 images in the dataset.

### 3.2. The DSCATNet

**Fig 1:**
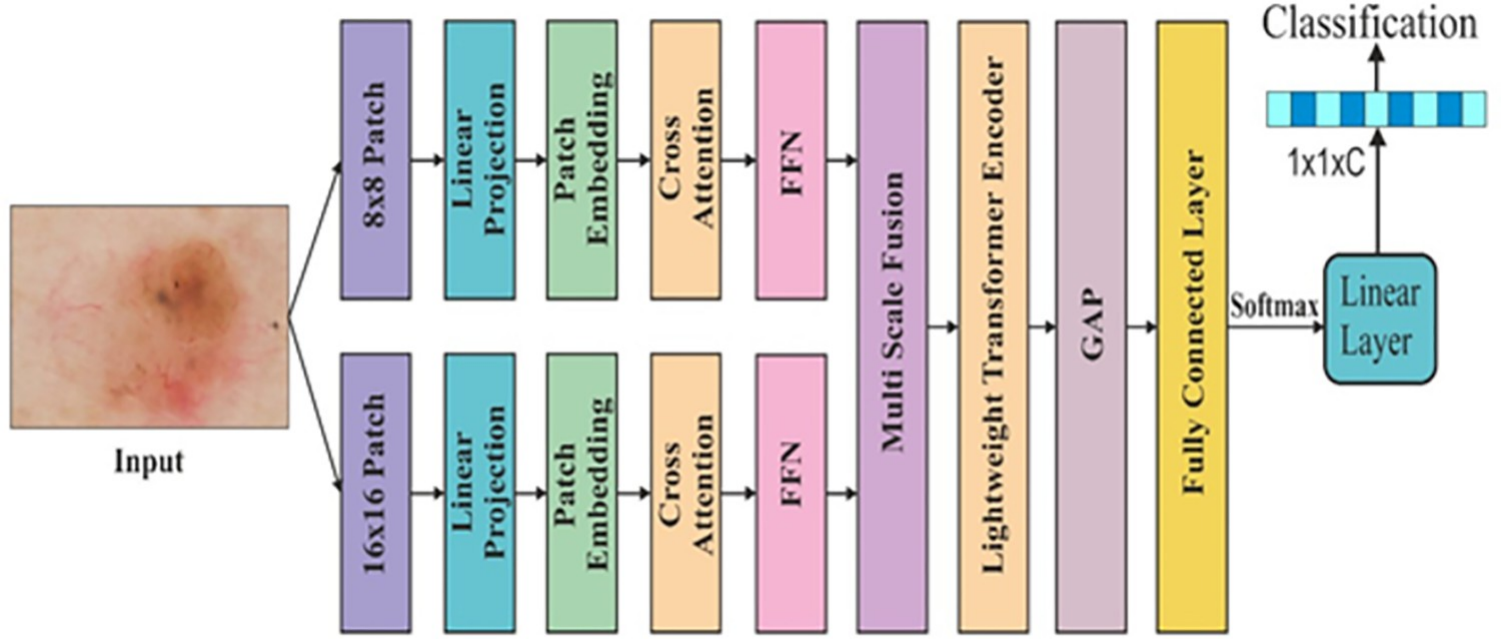
The architecture of the DSCATNet for skin lesion diagnosis.

In this study, we designed a dual-scale cross-attention transformer to diagnose the skin lesion. The input image is split into a patch size of 8x8 and 16x16 pixels. These patches are linearly projected, and embedding is performed. After that, cross-attention is performed so that the interaction between Query (Q), Key (K), and Value (V) can be better. After that, dual-scale attention heads are combined and fed to the transformer encoder. The encoder has LN (layer norm), MHCA (multi-head cross-attention), and FFN (feed-forward network). The LN provides the original contextual information to stabilize the training process. MHCA captures information on skin lesions on different scales. The FFN consists of two feed-forward neural networks and a ReLU activation function, which adds non-linearity to the model. Fig 1 shows the architecture of the DSCATNet.

Let the input image I∈R^H×B×C^, here H, B, and C are height, width and number of channels, respectively. From the input image, the patch for scale 1 and S_2_×S_2_ for scale 2 is extracted as follows.

$$I_{s}^{i}=\{I_{x,y}^{i}|x\in \{1,2,\ldots \frac{H}{S_{i}}\},y\in \{1,2,\ldots \frac{B}{S_{i}}\}\}$$ (1)

Where $I_{x,y}^{i}\in R^{S_{i}\times S_{i}\times C}$, x,y = Position of the row and column in the grid patch. The generated patches are flattened and projected to the embedding dimension of D; for scale 1 and scale 2, we set D = 192 and 768, respectively. For embedding the patches, we first multiplied flattened features with the weight matrix; then, bias is added. The complete embedding process is described in Eq (2).

$$E_{x,y}^{i}=W_{S_{i}}\cdot flatten(I_{x,y}^{i})+b_{S_{i}}$$ (2)

Where, $W_{S_{i}}\in R^{D\times (S_{i}\times S_{i}\times C)}$ and $b_{S_{i}}\in R^{D}$, $E_{x,y}^{i}$ = Embedded patch at position (x,y) of scale i. $W_{S_{i}}$ = Weight matrix. $I_{x,y}^{i}$ = Patch extracted from input at position (x, y) of scale i. $b_{S_{i}}$ = Bias added to the scale i. After that, these two scale-embedded patches are concatenated as follows.

$$M_{e}=[E^{1},E^{2}]+P_{o}$$ (3)

Where, P_o_∈R^N×D^ is the positional encoding N = Total patches, M_e_ = Multiscale position encoding. After position encoding, we extracted query (Q), key (K), and value (V) from each scale.

$$Q^{i}=W_{q}^{i}M_{e},K^{i}=W_{k}^{i}M_{e},V^{i}=W_{v}^{i}M_{e}$$ (4)

Where, $W_{q}^{i},W_{k}^{i},W_{v}^{i}\in R^{D\times D}$. From the Q of scale i and K from scale j, we calculated cross-attention at each pair of scales (i, j) as follows.

$$Att^{\left(i,j\right)}=\frac{Q^{i}K^{(j)T}}{\sqrt{D}}$$ (5)

Further, the Softmax function is applied to the raw attention to convert it into a weighted attention score, by doing this, the model focuses on the important features of the skin lesion region. This process is defined as follows.

$$W_{att}=Soft\max(Att^{\left(i,j\right)})$$ (6)

The weighted attention (W_att_) provides the importance of each value in V^j^ for the query Q^i^ and it is used to calculate the attention output (A_o_). This new representation of each query contains information from all the values.

$$A_{o}^{\left(i,j\right)}=W_{att}^{\left(i,j\right)}V^{j}$$ (7)

Furthermore, we calculated the cross attention and attention output of the head for each pair at two scales using Eqs (8) and (9). Here, head (h) = 12 and D = 192, 768 for scale 1 and scale 2, respectively.

$$Att_{h}^{\left(i,j\right)}=Soft\max(\frac{Q_{h}^{i}K_{h}^{(j)T}}{\sqrt{D/h}})$$ (8)

$$A_{oh}^{\left(i,j\right)}=Att_{h}^{\left(i,j\right)}V_{h}^{j}$$ (9)

After calculating the attention output, we concatenated them as follows.

$$MHCA^{\left(i,j\right)}=concat(A_{oh_{1}}^{\left(i,j\right)},A_{oh_{2}}^{\left(i,j\right)},\ldots ..A_{oh_{h}}^{\left(i,j\right)})W_{o}$$ (10)

Here, MHCA^(i,j)^ = Multi-head cross attention of pair (i,j). W_o_∈R^D×D^ = Weight matrix. The cross attention of the two scale is combined to generated enhanced attention using Eq (11).

$$Z_{1}=\sum_{i=1}^{S_{1}}\sum_{j=1}^{S_{2}}MHCA^{\left(i,j\right)}$$ (11)

**Fig 2:**
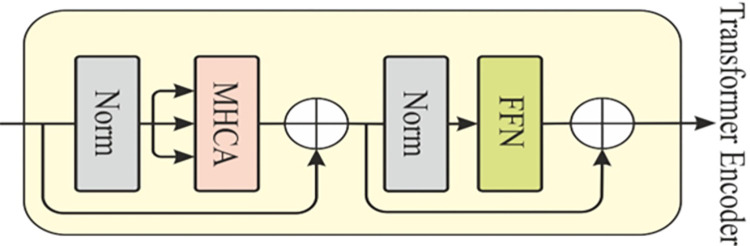
The architecture of the transformer encoder for skin lesion.

The normalization and feedforward operation in the encoder shown in Fig 2, is defined as follows.

$$\begin{array}{l} Z^{'}=LN(Z_{1}+M_{e}),\\ \tilde{Z}=FFN(Z^{'}),\\ Z_{r}=LN(\tilde{Z}+Z^{'}) \end{array}$$ (12)

Here LN = Layer Norm, FFN = Feed forward network, Z_r_ = Residual

The feature map obtained through the encoder is of variable length, which is converted to a fixed-size feature map using GAP (global average pooling). We also ensured that the reduction of spatial dimension doesn’t ignore the relevant information of the skin lesion image. The GAP is mathematically defined as follows.

$$GAP=\frac{1}{N}\sum_{i=1}^{N}Z_{r,i}$$ (13)

Here GAP∈R^D^, N = Number of tokens. On the top of the model, a fully connected layer is added, which converts the fixed-size feature map to the probability score of each class using the Softmax activation function as follows.

$$Y_{final}=Soft\max(W_{c}GAP+b_{c})$$ (14)

Where, Y_final_ = Final output, W_c_∈R^D×n^ = Weight matrix of the fully connected layer, b_c_∈R^n^ = Bias vector and n = Number of classes. We calculated the model of the loss using the categorical cross-entropy function on both datasets and it is defined as follows.

$$L_{cat}=-\frac{1}{BS}\sum_{i=1}^{BS}\sum_{j=i}^{C}y_{i,j}\log(P_{i,j})$$ (15)

Where, BS = batch size, C = Number of classes, y = True label and P = Predicated probability value j^th^ class in i^th^ sample. The variables of the proposed method is summarized in the Table 2.

**Table 2:** Summary of the variables used in the study.

Variables	Defination
x, y	Position of the row and column in the patch
D	Embedding dimension
W^i^	Weight matrix at scale i
P^i^(x,y)	Extracted path of the image at position(x,y) from scale i
b^i^	Bias at scale i
M_e_	Mutiscale positional encoding
Q^i^	Query at scale i
K^i^	Key at scale i
V^i^	Value at scale i
A(i,j)	Cross attention between query at scale i and key at scale j
Softmax(A(i,j))	Weighted attention score
h	Number of heads
Z_r_	Residual connection in the transformer
W_FC_	Weight matrix of the fully connected layer

The algorithm of the proposed method is as follows.

Algorithm 1: Algorithm for skin lesion diagnosis using DSCATNet

Input: I∈R^H×B×C^

Output: Classified label I∈R^1×1×C^

(1) Resize the input image to 224x224x3 pixels

(2) Set batch size = 32,initital learning rate = 0.001 and Epochs = 200

(3) Generate patch of 8x8 and 16x16 pixels

(4) Perform patch embedding $I_{s}^{i}=\{I_{x,y}^{i}|x\in \{1,2,\ldots \frac{H}{S_{i}}\},y\in \{1,2,\ldots \frac{B}{S_{i}}\}\}$ (5) Generate Q, K, V as follows $Q^{i}=W_{q}^{i}M_{e},K^{i}=W_{k}^{i}M_{e},V^{i}=W_{v}^{i}M_{e}$ (6) Calculate cross-attention for scale (i,j) as follows $Att^{\left(i,j\right)}=\frac{Q^{i}K^{(j)T}}{\sqrt{D}}$

(7) for i = 1 to 100 do

Train the model using 5-fold cross validation scheme

End

(8) Plot confusion matrix for each fold

(9) Plot the training loss curve

The component patch embedding requires O(N) time for N number of patches. Furthermore, the patch embedding requires O (dxS^2^xN), where S = Patch size, d = Patch embedding vector and N = Total number of patches. Moreover, the calculating Q, K and V requires O(Nxd^2^). Whereas, cross attention mechanism requires O(Nxd) time.

## 4. Experimetal results

In this section we presents the experimental results on HAM 10000 and PAD datasets.

### 4.1. Experimental settings

We experimented on Dell Precision 7920 Workstation, which has configuration as follows Intel Xeon Gold 5222 3.8 GHz Processor, Kingston 128 GB DDR4 2933 RAM, Kingston 1 TB 7200 RPM SATA HDD, Kingston 500 GB SSD, Nvidia Quadro RTX 4000 8GB Graphics Card, 24 Inch Dell TFT Monitor, Dell USB Mouse, Dell KB216 Wired Keyboard, Microsoft Windows 10 Operating System, Python 3.10 Programming Language, and Tensor Flow 2.0 open-source Machine Learning Framework. Furthermore, model scripting is written using Python 3.10. We set a batch size of 32 and an initial learning rate of 0.001 for all the experiments. The Adam optimizer initiates the training, and the model is trained for 200 epochs. The hyperparameters is summarized in Table 3.

**Table 3:** Summary of the hyperparameters.

Hyper parameters	Value
Batch size	32
Initial learning rate	0.001
Patch size	8x8 and 16x16
Optimizer	Adam
Epochs	200
Patch embedding dimension	192, 768

### 4.2. Performance evaluation on HAM10000 dataset

**Fig 3:**
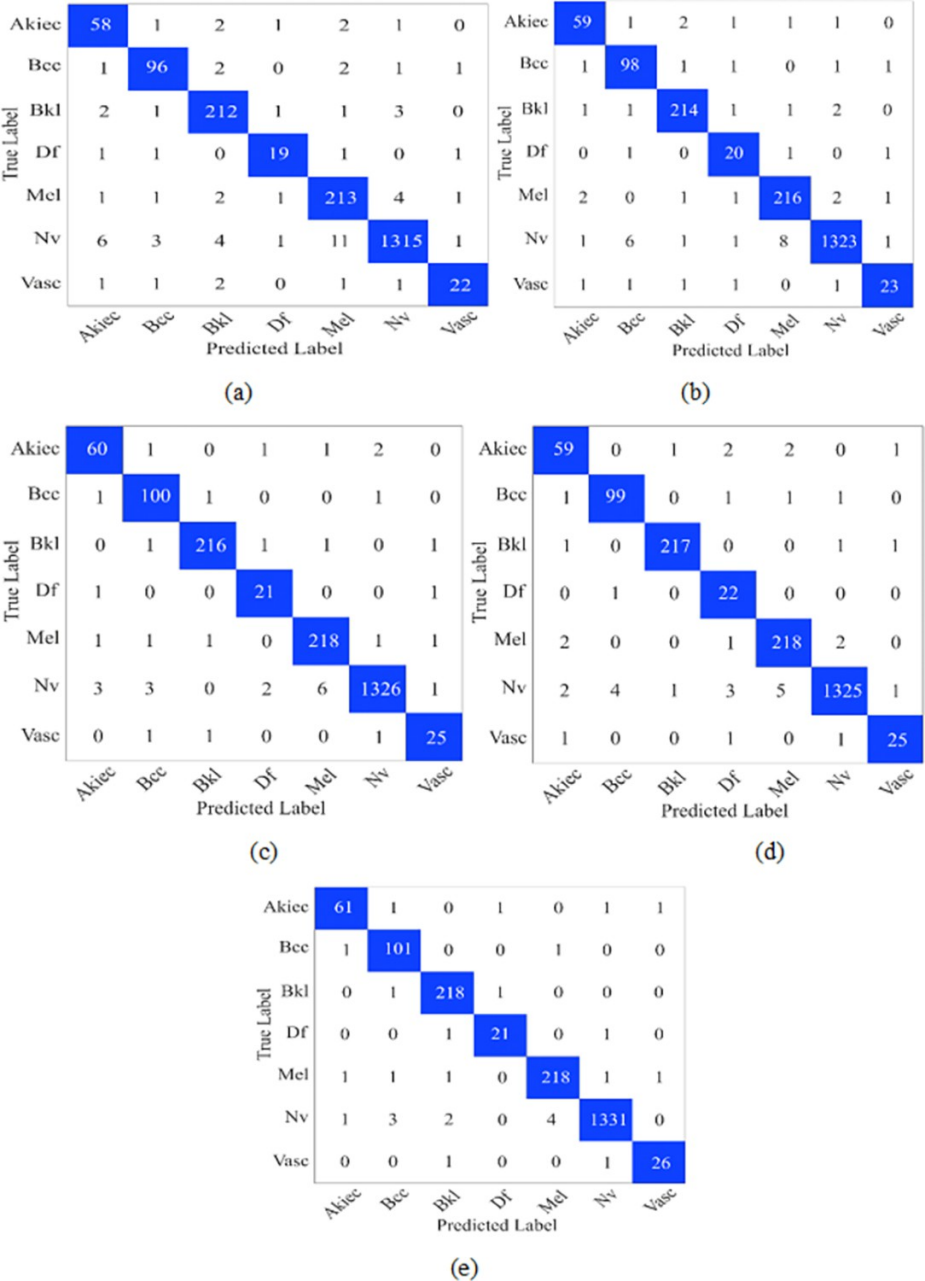
Confusion matrix on HAM 10000 dataset (a) Fold1 (b)fold2 (c) Fold3 (d) Fold4 and (e) Fold5.

In the HAM 10000 dataset each class contains unequal number of images. To avoid bias performance, we applied a 5-fold cross validation scheme. In a 5-folds cross validation scheme, we split the dataset into five equal folds. Out of these five fold one fold is used for validation and four fold for training. This process is repeated five times, and the model is trained for 200 epochs. The input image is resized to 224x224x3 pixels. After that, patches of size 8x8 and 16x16 is extracted for scale 1 and scale 2. Furthermore, on the patch, we performed embedding and trained the model using the Adam optimizer with a batch size of 32. Finally, we plotted the confusion matrices and presented in Fig 3.

In Fig 3(A), we can notice that the model has 26 FP (false positive) and 42 FN (false negative) values. Further, in fold 2, these values are decreased, and the FP value reached 20 and the FN value 30. Similarly, in other folds, these values are decreased. The morphological characteristics such as texture, color, and shape of the different classes present in the HAM 1000 and PAD datasets have very similar characteristics; due to this, the model has predicted FP and FN values. For example, Mel and NV classes have dark colors and irregular shapes, which can confuse the model. Similarly, BKL and AKIEC have very close rough textures and varied pigmentation. In addition, the BCC class can be misclassified as VASC due to visible blood vessels and MEL due to irregular pigments.

Finally, in Fold5, we have 9 FP and 18 FN values. Furthermore, performance metrics Kappa (K), recall (R), precision (P), F1-score (F), and accuracy (A) are calculated using the formula described in the literature [41]. Table 4 presents the performance measures calculated from the confusion matrix of the HAM 10000 dataset. In fold1 the kappa and F1 scores are 93.60% and 90.13%, respectively. At the same time, fold2 has precision and recall values of 92.54% and 90.79%, respectively. Moreover, fold 3 and fold 4 have precision values of 94.97% and 95.29%, respectively. Furthermore, the model’s average kappa and accuracy values on the HAM 10000 dataset are 95.84% and 97.80%, respectively.

**Table 4:** Performance metric on HAM 10000 dataset.

Fold	K (%)	F (%)	P (%)	R (%)	A (%)
Fold1	93.60	90.13	90.48	89.78	96.61
Fold2	95.30	91.66	92.54	90.79	97.50
Fold3	96.50	93.85	94.97	92.77	98.15
Fold4	96.40	93.49	95.29	91.76	98.10
Fold5	97.40	95.80	96.02	95.58	98.65
Average	95.84	92.98	93.86	92.14	97.80

### 4.3. Performance evaluation on PAD dataset

**Fig 4:**
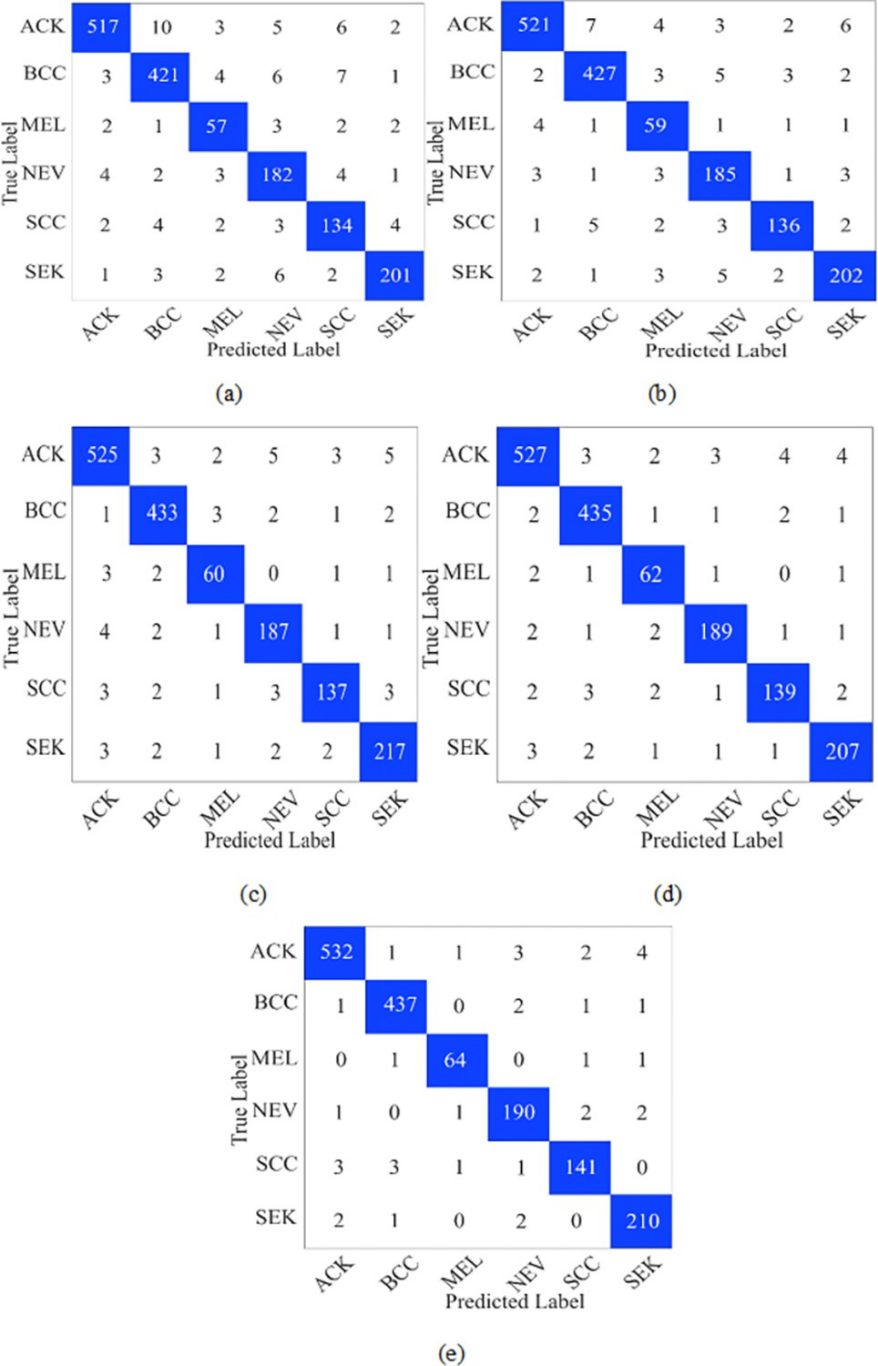
Confusion matrix on PAD dataset (a) Fold1 (b)fold2 (c) Fold3 (d) Fold4 and (e) Fold5.

This section presents the model’s results on the PAD dataset. The PAD dataset has less number of images in each class. Therefore, we applied the data augmentation technique and increased the image in each class 5 times. We also applied the same experimental setting and validation scheme described in section 4.1 for this dataset. The confusion matrices are presented in Fig 4. Fig 4(A) shows that the model has 60 FP and 40 FN values. Whereas in fold 2, these values are 44 and 38, respectively. Furthermore, in fold 3 and fold 4, decreases and lowest are in fold 5.

The performance metrics on the PAD dataset are depicted in Table 5. In fold 1 model achieved precision and accuracy values of 91.97% and 93.80%, respectively. At the same time, fold 2 has an F1-score and kappa value of 9276% and 93.43%, respectively. Furthermore, fold 4 and fold 5 have kappa values of 95.70% and 96.92%, respectively. Moreover, average kappa and accuracy are 94.56% and 95.81%, respectively, on the PAD dataset.

**Table 5:** Performance metric on PAD datasetand.

Fold	K (%)	F (%)	P (%)	R (%)	A (%)
Fold1	92.00	91.31	91.97	90.66	93.80
Fold2	93.43	92.76	93.37	92.16	94.91
Fold3	94.81	94.41	94.48	94.35	95.97
Fold4	95.70	95.42	95.67	95.18	96.71
Fold5	96.92	96.89	96.93	96.85	97.64
Average	94.56	94.16	94.49	93.84	95.81

## 5. Discussion

Skin cancer is a dangerous disease which requires treatment to save the patient’s life. Classical method of skin lesions diagnosis is time-consuming and expert-dependent. With the advancement in AI, automated systems have been developed to serve the second opinion of dermatologists. However, machine learning methods require hand-crafted texture, shape and colour features to train and test the algorithm. On the other hand, CNN-based methods automatically extract spatial features from the skin lesion and provide a reliable diagnosis. The CNN-based methods lack global attention to spatial features. In this study, we proposed DSCATNet, which has a dual-scale cross-attention module to focus on the relevant region of the skin lesion on a batch size of 8x8 and 16x16 pixels. Furthermore, the transformer encoder provides global contextual information to the spatial features. In the encoder, we designed MHCA to capture fine-grained spatial features from the different scales. We evaluated the model performance on the HAM 1000 and PAD datasets. In addition, the performance metric is compared with CNN and ViT-based methods to check the superiority.

### 5.1. Performance comparision on HAM 10000 dataset

In this section, we present the performance comparison of the proposed method with Inception V3 [42], ResNeXt [43], MobileNet [44], ViT [45] and SI-ViT [46]. The Inception V3, ResNeXtand MobileNet are convolution neural network models. The ViT and SI-ViT are vision transformer models. For a fair comparison, each model is trained and validated under the same experimental settings described in section 4.1. In Table 6, we can observe that Inception V3 has the second lowest precision and recall values of 78.24% and 81.06%, respectively; the ResNeXt has high-performance measures in all the CNN-based methods. Furthermore, the MobileNet showed the lowest kappa and F1 scores of 79.23% and 77.11%. However, transformer-based models have much better performance metrics compared to CNN-based methods. The ViT achieved 92.36% and 94.45% kappa and classification accuracy. Further improvement of kappa and accuracy can be noticed in the SI-ViT. Moreover, the proposed method has 95.84% and 97.80% kappa and classification accuracy on the HAM 10000 dataset.

**Table 6:** Performance comparison on the HAM 10000 dataset.

Method	K(%)	F(%)	P (%)	R (%)	A (%)
Inception V3	82.17	79.63	78.24	81.06	83.21
ResNeXt	88.79	86.71	86.14	87.28	90.32
MobileNet	79.23	77.11	77.65	76.57	80.97
ViT	92.36	90.85	90.42	91.29	94.45
SI-ViT	94.16	91.62	92.02	91.23	95.35
DSCATNet	95.84	92.98	93.86	92.14	97.80

### 5.2. Performnacecomparision on PAD dataset

In Table 7, we depicted the performance comparison of the proposed method withInception V3, ResNeXt, MobileNet, ViT and SI-ViT. All the experiments are performed with the same experimental settings described in section 4.1 for fair comparison. The performance metric of the Inception V3 is the lowest. The ResNeXt has kappa and F1-score of 85.17% and 83.90% respectively. Moreover, MobileNet has an F1-score and classification accuracy of 81.34% and 86.27%, respectively. The transformer-based method ViT and SI-ViT have 90.87% and 93.06% classification accuracy. Meanwhile, the proposed method has precision and accuracy of 94.49% and 95.81%, respectively.

**Table 7:** Perfomance comparision on the PAD dataset.

Method	K (%)	F (%)	P (%)	R (%)	A (%)
Inception V3	78.67	75.72	76.18	75.26	80.12
ResNeXt	85.17	83.90	83.29	84.72	87.45
MobileNet	84.26	81.74	81.34	82.15	86.27
ViT	87.98	85.89	85.56	86.24	90.87
SI-ViT	92.74	91.20	91.82	90.59	93.06
DSCATNet	94.56	94.16	94.49	93.84	95.81

### 5.3. Training loss

**Fig 5:**
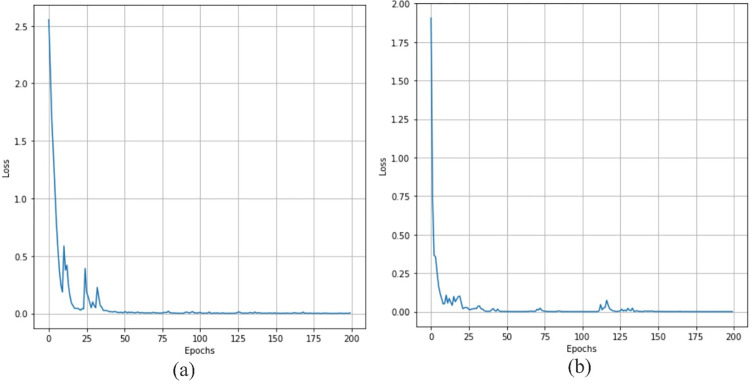
Training loss on (a) HAM 10000 dataset and (b) PAD dataset.

The training loss of the proposed model on the HAM 10000 and PAD datasetsare shown in Fig 5(A) and 5(B) respectively. In Fig 5(A), we can observe that the model’s training loss on the HAM 10000 dataset is initially more than 2.5. It starts gradually decreasing and reaches close to zero after 30 epochs. The initial model has a training loss of more than 1.75 on the PAD dataset. After 25 epochs, it starts decreasing and reaches near to zero after 126 epochs.

### 5.4. ROC (Receiver Operating Characteristic) based performance comparision

**Fig 6:**
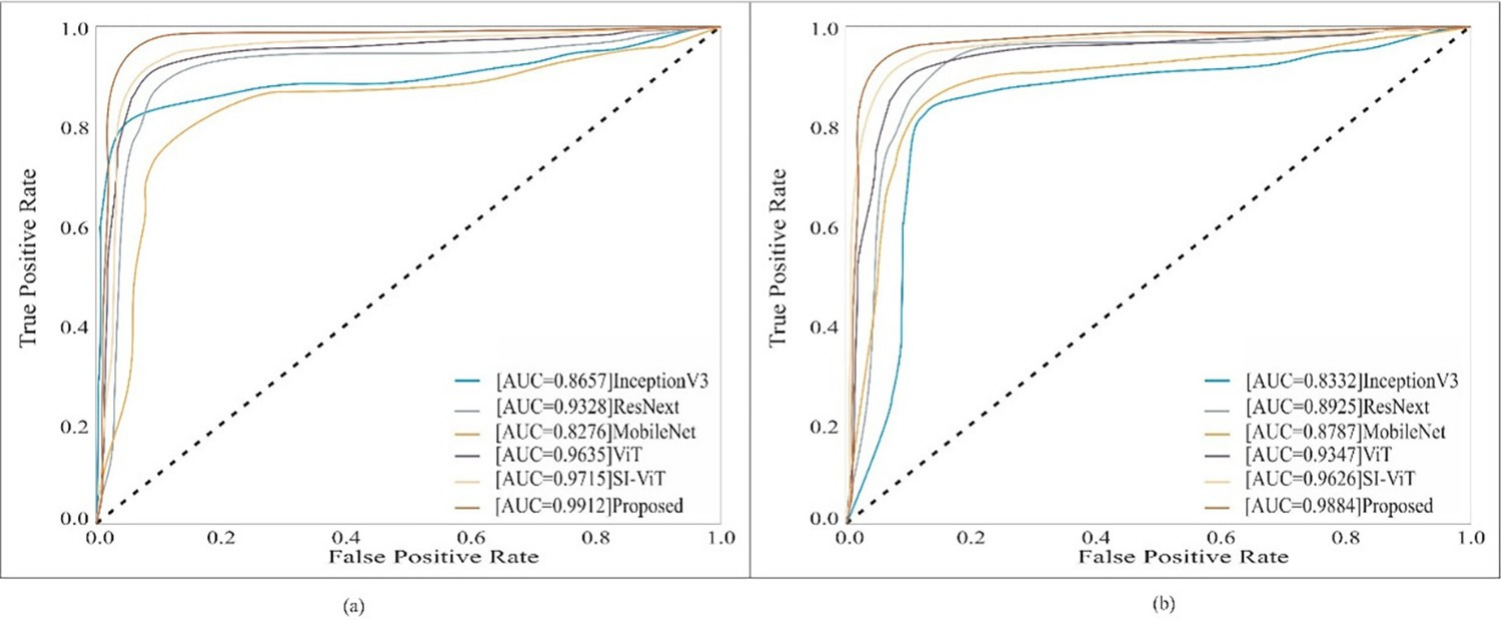
ROC plot on (a) HAM 10000 dataset (b)PAD dataset.

This section presents the ROC-based comparison of the proposed model withInception V3, ResNeXt, MobileNet, ViT, and SI-ViT on the HAM 10000 and PAD datasets, as shown in Fig 6. The ROC curve is plotted between true positive and false positive rate [47]. In Fig 6(A), the AUC (area under curve) values of the Inception V3 and MobileNet are 0.8657 and 0.8276, respectively. Whereas, ResNext has 0.9328. Furthermore, transformer-based models ViT and SI-ViT have more than 0.95 AUC values. Moreover, the proposed model has an AUC value of 0.9912 on the HAM 10000 dataset. On the PAD dataset, CNN-based methods, such as Inception V3, ResNeXt, and MobileNet, have an AUC value below 0.9. However, the transformer-based methods ViT and SI-ViT have AUC values of 0.9347 and 0.9626, respectively. Moreover, the proposed model achieved a 0.9884 AUC value.

### 5.5. Training and validation time comparision

The training and validation time of the proposed model and Inception V3, ResNeXt, MobileNet, ViT and SI-ViT on HAM 10000 and PAD dataset is shown in Table 8. We can notice that Inception V3 and MobileNet have less train and test time on both datasets due to their fewer trainable parameters. ResNeXt has takes more train and validation time due to its complex architecture of 50 layers and significant trainable parameters of 24x106. Furthermore, ViT and SI-ViT require high computation time due to the encoder’s self-attention calculation. The proposed method takes more train and test time than Inception V3 and MobileNet. Furthermore, our model has a closer computation time with ResNeXt and less than other transformer methods.

**Table 8:** Training and validation time comparison on HAM 10000 and PAD dataset.

Method	HAM10000		PAD	
	Train (m)	Val (s)	Train (s)	Val (s)
Inception V3	187	124	145	105
ResNeXt	216	157	203	143
MobileNet	151	104	134	114
ViT	242	173	226	157
SI-ViT	225	164	209	136
Proposed	212	149	196	125

### 5.6. Ablation study

In this section we present ablation study on patch size, which effect proposed model performance on HAM 10000 and PAD dataset.

#### 5.6.1. Effect of patch size

The patch size plays a crucial role in the transformer-based method. We present the different patch sizes on scale 1 (S1) and scale 2 (S2) on HAM 10000 and PAD dataset in Table 9. We notice small patch sizes of (4x4) and (4x4) on S1 and S2 have kappa and precision of 90.18% and 87.32%, respectively, on the HAM 10000 dataset. Furthermore, the same patch size of (8x8) on both scales improved the performance measures. Moreover, different-size patches on S1 (8x8) and S2 (16x16) achieved the highest kappa and precision values of 95.84% and 93.86%, respectively. Larger patch sizes increased the computation cost and decreased performance measures slightly. Similar trends can be observed in the PAD dataset. Smaller patch sizes have less accuracy and precision value. The same patch size on S1 and S2 slightly improved, and a larger patch size increased the computation costs.

**Table 9:** Performance metric under different patch size.

Dataset	Patch	K (%)	P(%)	R(%)	A(%)
HAM 10000	S1: (4x4), S2: (4x4)	90.18	87.32	88.56	92.67
	S1: (8x8), S2: (8x8)	92.34	91.06	91.49	94.73
	S1: (8x8), S2: (16x16)	95.84	93.86	92.14	97.80
	S1:(16x16), S2: (32x32)	93.12	92.02	90.87	96.23
PAD	S1: (4x4), S2: (4x4)	86.42	85.98	84.79	88.19
	S1: (8x8), S2: (8x8)	89.04	87.21	88.36	92.69
	S1: (8x8), S2: (16x16)	95.46	94.49	93.84	95.81
	S1:(16x16), S2: (32x32)	93.27	92.13	91.52	94.05

#### 5.6.2. Cross sensor based evaluation

The effectiveness of the DSCATNet is evaluated using cross-sensor data. The HAM 10000 dataset has seven categories of skin lesion images. In contrast, the PAD dataset has six classes of skin lesion images. The HAM 10000 and PAD datasets have four common skin lesion categories: ACK, BCC, MEL and NEV. Furthermore, the HAM 10000 has 10015 and the PAD dataset has 1612 skin lesion images. We trained our model on the HAM 10000 dataset and validated it on the original 1612 images of the PAD dataset under the same experiment described in section 4.1. The performance measures such as kappa precision, recall, F1-score and accuracy are shown in Table 10. Table 10 shows that the model achieved good kappa and precision scores of 93.27% and 91.17%, respectively. In addition, classification accuracy is 94.18%. This confirms that common morphological characteristics such as the skin lesion colour, shape and texture are crucial for better diagnosis.

**Table 10:** Cross dataset-based performance comparison.

K (%)	F (%)	P (%)	R (%)	A (%)
93.27	91.17	90.82	91.52	94.18

### 5.7. Performance evaluation on the histopathological image dataset

**Fig 7:**
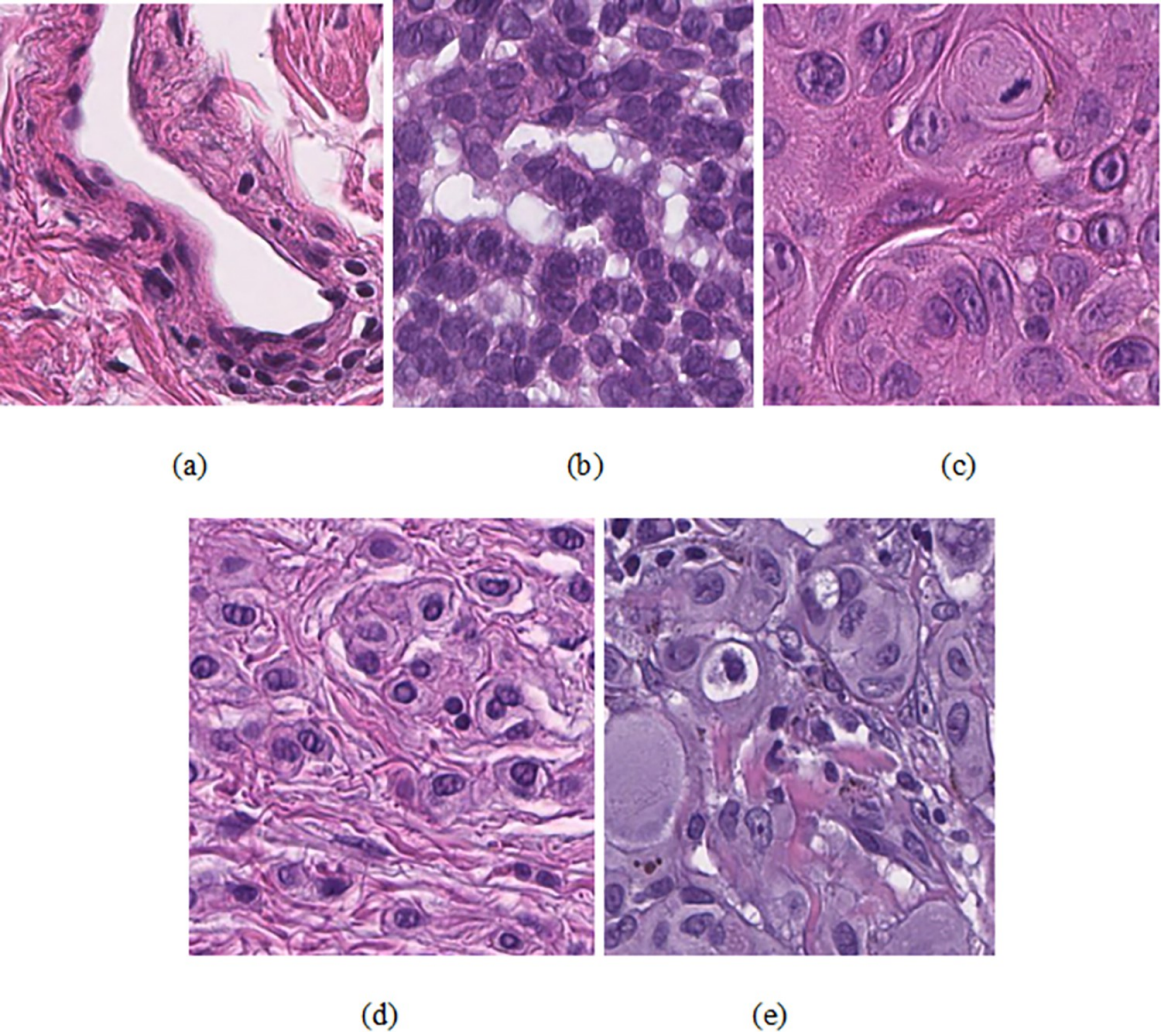
Sample images (a) Vessels (b) BCC (c) SqCC (d) Naevi and (e) Melanoma.

In this study, we utilized two datasets, HAM10000 and PAD dataset, which are very diverse. However, for further model effectiveness, we conducted an experiment on the histopathological images [48]. This dataset has 129364 images categorized into 16 classes from 386 cases. The dataset is manually annotated, and the images have a resolution of 395x395 pixels. Due to hardware constraints, we created a new dataset of the 44168 images from the mentioned dataset. The new histopathological images dataset has five classes: Vessels, BCC, SqCC, Naevi and Melanoma. The summary of the train and test image is shown in Table 11, and some sample images are in Fig 7.

**Table 11:** Data distribution in each class of the histopathological image dataset.

Class	Total	Train	Val
Vessels	1752	1402	350
BCC	8923	7139	1784
Naevi	10629	8503	2126
SqCC	11182	8946	2236
Melanoma	11682	9346	2336

**Fig 8:**
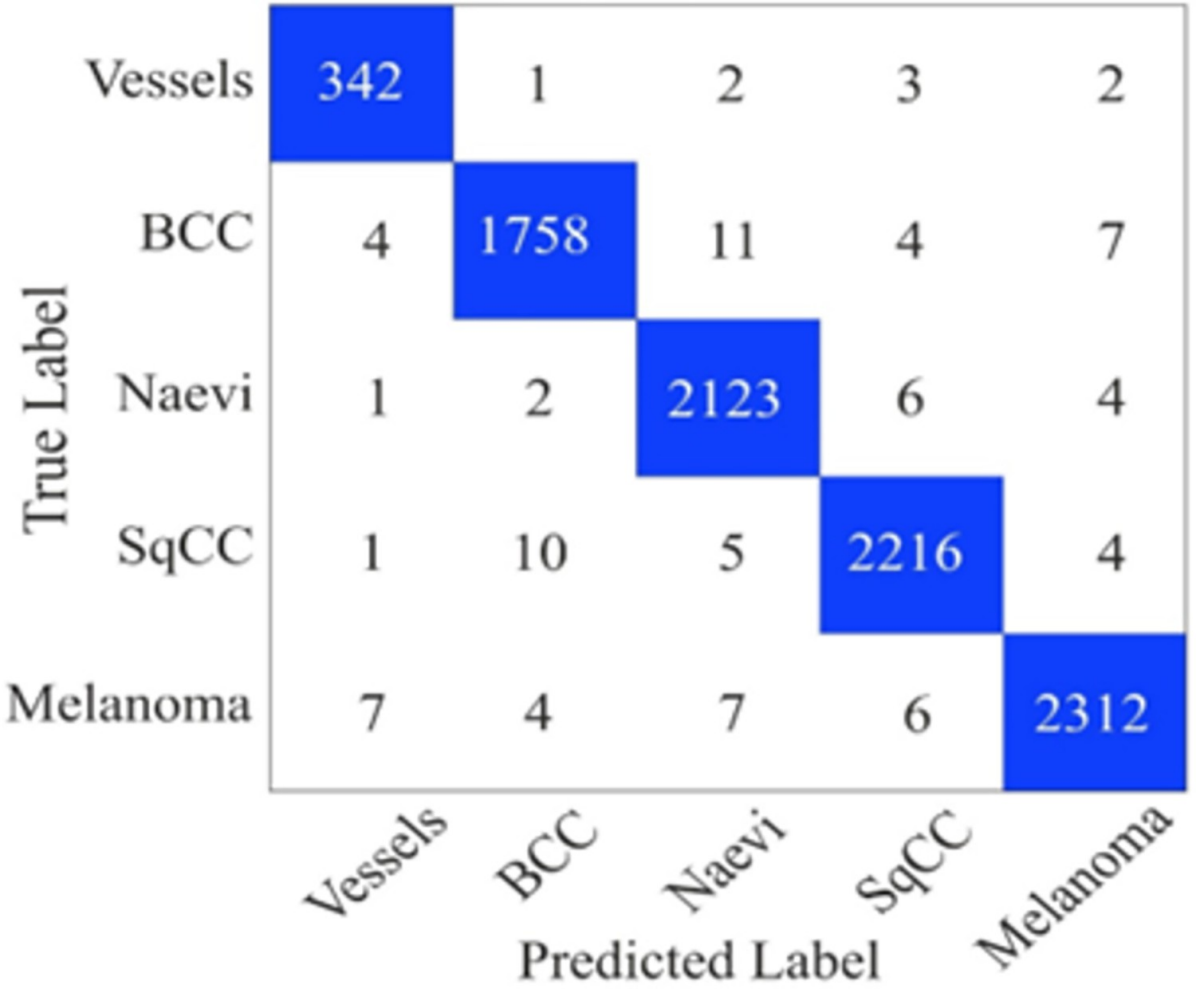
Confusion matrix on the histopathological image dataset.

The proposed dataset has 44168 images stored in Jpeg format. We randomly divided the dataset into 80% and 20% for the training and validation. After that, the model is trained for 75 epochs using the Adam optimizer in a batch size of 32. The confusion matrix of the proposed model on the dataset is shown in Fig 8. In Fig 8, we can observe that the model has 45 FP and 47 FN values.

From the confusion matrix, we calculated the performance measures precision, recall, F1-score, accuracy and Kappa shown in Table 12. We can see that the model achieved 98.70% kappa value and 98.52% precision.

**Table 12:** Performance measures on the histopathological image dataset.

K (%)	F (%)	P (%)	R (%)	A (%)
98.70	97.13	96.74	98.52	98.97

**Fig 9:**
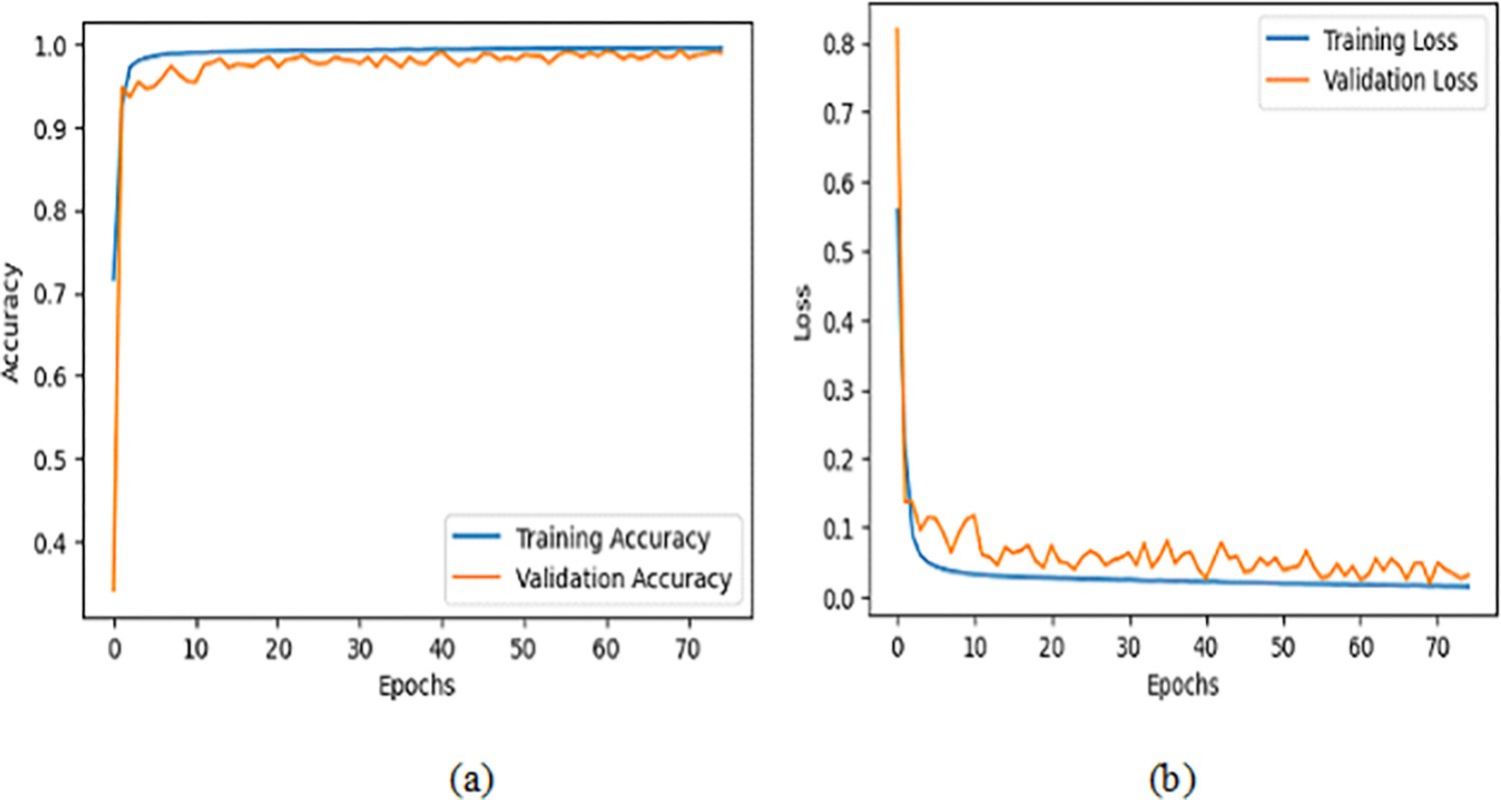
The training and validation accuracy and loss of the proposed model.

Furthermore, we plotted the proposed model’s training and validation loss and accuracy curves, as shown in Fig 9. We can observe in Fig 9(A) that initially, training and validation loss is less. However, after 20 epochs, it reached more than 98%. Moreover, Fig 9(B) shows an initial training loss of more than 0.5. However, after 15 epochs, it reached below 0.1.

### 5.8. Effect of hyperparameters on model performance

The proposed DSCATNet has two-scale cross-attention and MHCA (multi-head cross-attention) in the encoder block. In addition, hyperparameters, such as the number of head selection and embedding dimensions, play crucial roles in model performance. We experimented with the heads H = {4, 8, 12, 16} and found that a smaller head value (4) was not able to achieve high performance. The increase in the head value improved the performance, and the model achieved the best accuracy with a head value 12. In addition, embedding dimension selection is also important for good classification performance. We experimented with the embedding dimension D = {128, 192, 256, 512, 768, 1024}, and we found that a higher embedding dimension requires high computation costs, and an overfitting problem arises. However, with a lower embedding dimension, it could not achieve high performance. The proposed model achieved good skin classification accuracy by embedding dimensions 192 and 768 at scale1 and 2. After setting parameters, we compared the DSCATNet performance on the HAM 10000 dataset with classical ViT, as shown in Table 12. Table 13 shows that ViT with MHSA (multi-head self-attention) has a precision value of 90.42%. However, the inclusion of the two-scale cross-attention improved the precision by 1.15%. Moreover, our model achieved a 93.86% precision value.

**Table 13:** Performance comparison with ViT on HAM 10000 dataset.

Model	K (%)	F (%)	P (%)	R (%)	A (%)
VIT+MHSA	92.36	90.85	90.42	91.29	94.45
ViT+ MHSA+Two scale Cross Attention	93.17	90.86	91.57	91.56	95.23
DSCATNet + MHCA+Two scale Cross Attention	95.84	92.98	93.86	92.14	97.80

In the proposed study, we tested our model on two diverse datasets. However, we must test our model on the skin lesion images captured through devices, including mobile phones and cameras, that may impose noises and artefacts in large volumes in real-time clinical practice. The proposed DSCATNet has a dual-scale cross-attention block, which can reduce the noise and artefacts from the images. In addition, the transformer encoder provides global attention to the spatial features. However, there may be slight differences in the performance in real-time clinical datasets.

## 6. Conclusion

In this study, we designed a dual-scale cross-attention transformer to diagnose the skin lesion. Our method extracts two patches of size 8x8 and 16x16 for dual scale, and cross attention is utilized to focus on the different regions of the skin lesion. Furthermore, a feature fusion module merges the spatial features. These features are fed to the encoder block for global co-relation and extract relevant features from the skin lesion images and the model is evaluated on the HAM 10000 and PAD dataset using a 5-fold cross-validation scheme. The DSCATNet achieved 97.80% and 95.84% classification accuracy and kappa score on the HAM 10000 dataset. The model obtained a precision and accuracy of 94.49% and 95.81%, on the PAD dataset. More lightweight architecture can reduce the dual scale’s multi-head attention computation costs. In addition, the computation costs of the attention mechanism need further refinement. The model needs to be tested on a real-time diverse dataset so that its effectiveness can be further validated. In future studies, we will add more layers to the transformer encoder for better classification performance. In addition, multi-scale transformers can be designed for more enhanced spatial features. Furthermore, nature-inspired algorithms can be used to optimize the spatial features.
